# Quality Comparison of Biosimilar and Copy Filgrastim Products with the Innovator Product

**DOI:** 10.1007/s11095-018-2491-5

**Published:** 2018-10-02

**Authors:** Liem Andhyk Halim, Maripaz Márquez, Roel F. Maas-Bakker, Gilberto Castañeda-Hernández, Wim Jiskoot, Huub Schellekens

**Affiliations:** 1 Department of Pharmaceutics, Utrecht Institute for Pharmaceutical Sciences (UIPS), Utrecht University, Universiteitsweg 99, Room 3.76, 3584 CG Utrecht, The Netherlands; 2 Departamento de Farmacología, Centro de Investigación y de Estudios Avanzados del Instituto Politécnico Nacional (Cinvestav), Av. IPN 2508, Col. San Pedro Zacatenco, C.P, 07360 Ciudad de México, Mexico; 3 Division of BioTherapeutics, Leiden Academic Centre for Drug Research, Leiden University, P.O. Box 9502, 2300 RA Leiden, The Netherlands

**Keywords:** biosimilar, copy product, filgrastim, protein characterization, quality

## Abstract

**Purpose:**

Filgrastim, a recombinant human granulocyte-colony stimulating factor, is widely used to treat congenital and acquired neutropenia. Following patent expiration of the innovator filgrastim product, biosimilar filgrastim products have been approved in the EU and shown to be comparable with the innovator with respect to quality, safety and efficacy. In less regulated markets, copy filgrastim products are available but data about their quality are scarce. In the present study, we provide a head-to-head comparative study on the quality of biosimilar and copy filgrastim products.

**Methods:**

Innovator filgrastim product, Neupogen®, two EU-licensed biosimilars, Zarzio® and Tevagrastim®, and two copy filgrastim products, Biocilin® and PDgrastim®, were subjected to peptide mapping, circular dichroism spectroscopy, fluorescence spectroscopy, sodium dodecyl sulfate polyacrylamide gel electrophoresis, high performance size-exclusion chromatography, reversed-phase ultra-performance liquid chromatography, endotoxin test, flow imaging microscopy and *in vitro* potency assay.

**Results:**

Zarzio® and Tevagrastim® have comparable quality to Neupogen®, while Biocilin® showed a significantly lower and PDgrastim® a higher specific activity. Moreover, PDgrastim® showed a higher level of impurities and a lower thermo stability than the other products.

**Conclusions:**

Except for the deviating specific activities of the two copy filgrastim products, we found no substantial differences in product quality between the filgrastim products studied.

## Introduction

Recombinant human granulocyte-colony stimulation factor (rhG-CSF) is one of the first recombinant biologics that was authorized for the use in hematology (1). Two different forms of rhG-CSF are distinguished, namely filgrastim (from *Escherichia coli* [*E. coli*], under the trade name Neupogen®) and lenograstim (from Chinese hamster ovary cell, brand name Granocyte®) (2). Filgrastim contains 175 amino acids and differs from the endogenous protein, as it lacks O-glycosylation and has an additional methionine group at the N-terminus (3). Its use was approved in 1991 in the EU and US. A next generation filgrastim is pegfilgrastim (brand name Neulasta®). Owing to the covalent attachment of 20 kDa polyethylene glycol (PEG) to the N-terminal methionyl residue of filgrastim, pegfilgrastim has extended circulating half-life, and thus can be administered less frequently than filgrastim (4).

RhG-CSF is commonly prescribed to treat both congenital and acquired neutropenia, such as in patients undergoing chemotherapy or patients diagnosed with advanced AIDS (5–7). Neutropenia is characterized by a low neutrophil count making patients more prone to bacterial infections. In addition, rhG-CSF is usually administered to donors of peripheral blood stem cells and progenitor cells prior to harvesting (8).

Patent expiration of innovator biologic products has created the possibility for companies to develop biosimilars. Within the EU, according to the European Medicines Agency (EMA), biosimilars are authorized via an abbreviated regulatory pathway, which requires demonstration of similarity in terms of quality, safety and efficacy, to an innovator product already licensed in the EU (9). Many countries are introducing specific biosimilar regulations using the European approach as guidance, but adapted to local needs and demands (10). In the US, the Biologics Price Competition and Innovation of 2009 (BPCI Act) enabled an abbreviated regulatory pathway for biosimilars. The Food and Drug Administration (FDA) issued a series of (draft) guidances, describing the quality, safety and efficacy data needed to show similarity between the candidate biosimilar to the reference product. The FDA takes the approach of totality of evidence in assessing that no clinically meaningful differences in safety, purity and potency exist between biosimilar and original product (10,11). In some regions, the approval of copy versions of biologics predates the introduction of biosimilar guidelines (12). Because these cannot be called biosimilars, a myriad of terms has been used such as “intended copies”, “non-original biologics” or “non-innovator biologics”, “similar biologics” (India), “biogenerics” (Iran) and “biocomparables” (Mexico) (10,13).

Biologics, as opposed to chemically-synthesized small molecule drugs, are extremely intricate and can undergo multiple degradation processes. Thereby, its characterization remains a challenge. The use of living cells may introduce subtle differences despite using the same gene sequence and vector as the innovator. All biologics are subjected to post­translational modifications, such as deamidation and oxidation, which may affect protein function and result in heterogeneity.

Product-related quality differences may in principle lead to serious adverse events. An often cited incident occurred in 1998 for the cases of Epoetin-associated Pure red cell aplasia (PRCA) in renal failure patients, where the change in the formulation from Human Serum Albumin to polysorbate 80, subcutaneous administration and uncoated robber stoppers have been involved with the presence of anti-drug and neutralizing endogenous erythropoietin antibodies (14). The high prevalence of PRCA in Thailand has been related with the bio-questionable quality of some recombinant human erythropoietin copy products (15). These are clear examples of how subtle changes in manufacturing process and quality properties may have unforeseen clinical consequences.

Currently, seven biosimilar filgrastim products have been approved in the EU (16). One product has successfully entered the US market as the first US biosimilar, Zarzio® (17). Zarzio has proved in US after shown highly similarity in terms of physicochemical properties, biological characterization, pharmacodynamic and pharmacokinetic parameters (18). All demonstrate comparable quality, safety and efficacy to Neupogen®, as reported in several comparative studies (1,18–22). In less regulated markets, different copies of filgrastim products have also been identified. In Mexico, selected filgrastim biocomparables complied with the pharmacopoeia criteria and showed comparability in terms of quality (23). In India, several copy filgrastim products showed similarity to Neupogen® with respect to physicochemical and biological characteristics (24,25). In Egypt, the quality of one copy filgrastim product was reported to be inferior to that of the innovator product (26). However, there is scarcity of data regarding direct comparison between the biosimilar and copy filgrastim products to the innovator product. The perceptions of clinicians is that copy biologics from less regulated markets are inferior to biosimilars, as these products have not been approved through a stringent regulatory process (27).

Here, we present a head-to-head comparison based on the evaluation of physicochemical properties and *in vitro* potency of two biosimilars approved in Europe and USA (Zarzio® and Tevagrastim®) and two copy products of filgrastim available in developing countries (Biocilin® and PDgrastim®), using as a reference the innovator product (Neupogen®). To encourage the manufacturers in developing countries to perform a complete analytical and functional head-to-head comparison between the copy product and the innovator, we used selected techniques sensitive, feasible and easy access mainly available in Ph. Eur.

## Materials and Methods

### Filgrastim Products

Table I lists innovator, biosimilar and copy filgrastim products procured from various sources. Different batches of Neupogen were either provided by Sandoz or purchased from the pharmacy at the University Medical Centre Utrecht (UMCU), Utrecht, The Netherlands. Biosimilar filgrastim products, Zarzio and Tevagrastim, were provided by the pharmacy of the UMCU. Copy filgrastim products, Biocilin and PDgrastim, were locally procured from Mexico and Iran, respectively, and shipped to Utrecht University. All products were stored at 2–8°C and handled according to the manufacturers’ specifications. Expired vials of Neupogen and PDgrastim were analyzed as a worst case scenario for marketed products.

**Table I:** List of Tested Filgrastim Products

Trade name (company)	Lot. no.	Declared potency		Declared content	Excipients	Presentation
Analyzed within shelf life						
Neupogen® (Amgen)	1042036A 1056681	30 MU/0.5 ml 48 MU/0.5 ml		300 μg/0.5 ml 480 μg /0.5 ml	Acetate, sorbitol, polysorbate 80 (PS80)	PFS
Zarzio® (Sandoz)	46081101	30 MU/0.5 ml		300 μg/0.5 ml	Glutamate, sorbitol, PS80	Vial
Tevagrastim® (Teva Pharma)	FL5028G	30 MU/0.5 ml		300 μg/0.5 ml	Glacial acetic acid, sorbitol, PS80	PFS
Biocilin® (Dong-A-Pharmaceutical)	40168	Not declared		300 μg/1.2 ml	Not stated	Vial
Analyzed past the expiry date						
Neupogen® (Amgen)	1025277 1026361 1026690 1026689 1027991 1028686 1028687	(A) (B) (C) (D) (E) (F) (G)	48 MU/1.6 ml 48 MU/1.6 ml 48 MU/1.6 ml 48 MU/1.6 ml 48 MU/1.6 ml 48 MU/1.6 ml 48 MU/1.6 ml	480 μg/1.6 ml 480 μg/1.6 ml 480 μg/1.6 ml 480 μg/1.6 ml 480 μg/1.6 ml 480 μg/1.6 ml 480 μg/1.6 ml	Acetate, sorbitol, PS80	Vial Vial Vial Vial Vial Vial Vial
PDgrastim® (PooyeshDarou)	900–3­4		30 MU/1.0 ml	300 μg/1.0 ml	Not stated	Vial

Different batches of Neupogen are represented by alphabet (A) to (G)

PFS: pre-filled syringe

### Visual Inspection

Prior to any measurements, all formulations were visually assessed at the lab bench to check for the presence of visible particulates.

### Peptide Mapping

The primary structural similarities between products were confirmed by peptide mapping in accordance with European Pharmacopeia (Ph. Eur.) monograph for filgrastim concentrated solution (28). Filgrastim chemical reference substances (CRS) with lot number 2, purchased from the European Directorate for the Quality of Medicines and HealthCare (EDQM, Strasbourg, France) and filgrastim samples were pre-concentrated by using Amicon® Ultra-0.5 ml centrifugal filters with a cut-off of 10,000 NWML (Merck Millipore, Amsterdam, The Netherlands) following the manufacturer’s recommendations. In short, 125 μg of protein from each sample and standard was loaded to pre-washed filter devices. Retained protein was washed three times with Milli-Q water and the concentrated solute was recovered.

Endoproteinase Glu-C from *Staphylococcus aureus* V8 (Sigma-Aldrich, Zwijndrecht, The Netherlands), which selectively cleaves C-terminal peptide bonds at glutamic acid and aspartic acid residues, was prepared at a protein-to-enzyme ratio of 1:10. Subsequently, 20 mM sodium phosphate buffer pH 8.0 (Sigma-Aldrich) was added to a final volume of 100 μl and digestion was carried out at 37°C for 17 h. Peptides were separated by using reversed-phase chromatography on a XSelect® CSH™ C18 3.5 μm, 4.6 × 150 mm column (Waters Corporation, Milford, Massachusetts, USA) installed in a Waters 2695 Separations Module equipped with a Waters 2487 Dual λ Absorbance UV detector set at 215 nm. The column was pre-equilibrated at 60°C with 97%A (0.05% TFA in 5% ACN in Milli-Q water) and 3%B (0.05%TFA in 95%ACN in Milli-Q water) for minimally 2 h. Separation was achieved by applying the following linear gradients at a flow rate of 0.2 ml/min: 3%B→6%B (0–8 min), 6%B→34%B (8–25 min), 34%B→90%B (25–40 min), 90%B (40–45 min), 90%B→3%B (45–46 min), 3%B (46–75 min). Zarzio was not included due to lack of sample.

### Circular Dichroism (CD) Spectroscopy

Circular Dichroism (CD) spectra were acquired with an Olis® Rapid-Scanning Monochromator 1000 (On-Line Instrument System, Bogart, Georgia, USA). Thermal stability experiments were performed at 25, 55°C, i.e., below the melting point (~60°C) and 70°C on 300 μg/mL filgrastim samples, except Biocilin (250 μg/ml). A 0.1 cm pathlength SUPRASIL grade quartz cell (Hellma Analytics, Müllheim, Germany) was used to record data between 180 and 260 nm with a 1 nm sampling interval and an integration time of 10 s. The baseline spectrum was subtracted from each sample spectrum. As not all excipients of filgrastim products are specified by the manufacturers, Neupogen placebo buffer (50 mg/ml sorbitol, 0.04 mg/ml PS 80, 0.59 mg/ml sodium acetate and 0.035 mg/ml sodium chloride) was used to obtain the baseline spectrum. Zarzio was not included due to lack of sample. Data were expressed as the mean residual molar extinction coefficient (∆ε). All chemicals were purchased from Sigma-Aldrich (Zwijndrecht, The Netherlands).

### Fluorescence Spectroscopy

Fluorescence spectra were obtained with an FP-8300 fluorescence spectrometer (JASCO, IJsselstein, The Netherlands). Tertiary structure and thermal stability of filgrastim were evaluated based on the intrinsic fluorescence, by using a temperature ramp from 25°C to 70°C with 5°C increments. Filgrastim samples were kept at 300 μg/ml, except Biocilin (250 μg/ml). A 0.3 cm pathlength SUPRASIL grade quartz cell (Hellma Analytics, Müllheim, Germany) was used to record the emission spectra between 290 and 450 nm with a 0.5 nm sampling interval, an excitation wavelength of 280 nm, emission and excitation bandwidths of 5 nm and a scan speed of 500 nm/min. The baseline spectrum was subtracted from each sample spectrum. As not all excipients of filgrastim products are specified by the manufacturers, Neupogen placebo formulation (50 mg/ml sorbitol, 0.04 mg/ml PS 80, 0.59 mg/ml sodium acetate, and 0.035 mg/ml sodium chloride) was used to obtain the baseline spectrum. Zarzio was not included due to lack of sample. Data were expressed as fluorescence intensity in function of temperature. All chemicals were purchased from Sigma-Aldrich (Zwijndrecht, The Netherlands).

### Sodium Dodecyl Sulfate Polyacrylamide Gel Electrophoresis (SDS-PAGE)

SDS-PAGE was performed under non-reducing and reducing conditions by using Bolt® 4–12% Bis­Tris Plus Gels (10 wells), installed on a Bolt® Mini Gel Tank. Prior to loading, filgrastim samples were mixed with 1× Bolt® LDS Sample Buffer and heated for 5 min at 95°C. On each well, 50 ng of protein was loaded. Under reducing condition, 1× Bolt® Sample Reducing Agent was added. PageRuler Plus Prestained Protein Ladder, 10–250 kDa, was used as a molecular weight marker. Filgrastim CRS (EDQM) was used as a control. The running condition was set to a constant voltage of 165 V for 45 min in 1× Bolt® MOPS SDS running buffer. Proteins were visualized by using a SilverQuest™ Silver Staining Kit according to the manufacturer’s specifications. All materials were ordered from Thermo Scientific (Landsmeer, The Netherlands).

### High Performance Size-Exclusion Chromatography (HP-SEC)

Following the Ph. Eur. monograph for filgrastim concentrated solution (28), HP-SEC was performed on a 5 μm, 300 × 7.8 mm TSK­gel® G3000SW_XL_ column (Tosoh Bioscience, Griesheim, Germany) with a SecurityGuard Universal Guard Cartridge System as a pre­column (Phenomenex®, Utrecht, The Netherlands). Both pre-column and column were installed in a Waters 2695 Separations Module (Waters Corporation, Milford, Massachusetts, USA) equipped with Waters 2487 Dual λ Absorbance UV and Waters 2475 Multi λ fluorescence detectors. UV detection was performed at 215 nm and 280 nm. For fluorescence detection, excitation was performed at 280 nm, and the emission was recorded at 340 nm. A mobile phase containing 50 mM ammonium hydrogen carbonate (Sigma-Aldrich), adjusted with phosphoric acid (Acros Organics, Geel, Belgium) to pH 7.0, and filtered through a 0.45 μm nylon filter (Sartorius Stedim, Göttingen, Germany), was used. Prior to injection in triplicate, all filgrastim products were diluted to a concentration 0.25 mg/ml in 100 mM sodium acetate, adjusted with acetic acid (Merck, Darmstadt, Germany) to pH 5.0. Isocratic separation took place at a flow rate of 0.5 ml/min for 30 min. At the beginning of each experiment, 50 μl of 1 mg/ml bovine serum albumin (Sigma-Aldrich) in dilution buffer was injected into the column to reduce non-specific adsorption. The temperature of sample and column was maintained at 20°C and 30°C, respectively. A calibration curve based on monomer peak area was constructed by using seven concentrations of filgrastim CRS in the range of 0.05–1 mg/ml and used to calculate the concentration of filgrastim monomer in all products. Analysis was performed with Empower 3 software version 7.21.00.00.

### Reversed Phase Ultra-Performance Liquid Chromatography (RP-UPLC)

Oxidized forms of filgrastim, to be used as standards for RP-UPLC, were obtained according to the Ph. Eur. monograph for filgrastim concentrated solution (28). Briefly, an aliquot of 100 μl of 0.5 mg/ml filgrastim CRS was treated with 3 μl of 30% hydrogen peroxide (Merck) and incubated at 25°C for 15 min before adding 0.8 mg of L-methionine (Sigma-Aldrich). Reduced forms were produced by adding 0.125 mg dithiothreitol to 100 μl of 0.5 mg/ml filgrastim CRS and incubated at 35°C for 60 min.

RP-UPLC was performed on an Acquity Ultra Performance LC system (Waters Corporation) where a UPLC Acquity BEH300 C4 column (1.7 μm, 2.1 × 50 mm) was installed. The column was equilibrated at 60°C with 85% mobile phase A (0.1% trifluoroacetic acid [TFA, Sigma-Aldrich] in Milli-Q water) / 15% mobile phase B (0.1% TFA in 90% HPLC grade acetonitrile [Biosolve, Valkenswaard, The Netherlands]) until a stable baseline was reached. Separation was achieved by applying the following linear gradients at a flow rate of 0.25 ml/min: 15% B (0–0.5 min), 15% B-75% B (0.5–10.5 min), 75% B-15% B (10.5–11 min) and 15% B (11–11.5 min). While being maintained at 20°C, 1.5 μg of each filgrastim product was injected in triplicate. Detection took place with an Acquity™ PDA detector (Waters Corporation) at 215 nm.

### Endotoxin Test

Endotoxin concentration in filgrastim products was quantified with an endpoint chromogenic Limulus amebocyte lysate (LAL) QCL­1000™ assay, as described by the manufacturer (Lonza, Basel, Switzerland). Briefly, each filgrastim product was diluted in LAL reagent water at ratio 1:10, 1:20 and 1:40. 25 μl of each dilution was transferred to each well of pre­warmed LAL reagent grade multi­well plate at 37°C in duplicate. Hereafter, an equal volume of LAL was added to each microplate well, followed by gently tapping on the side to facilitate mixing, and the mixture was incubated at 37°C for another 10 min. 50 μl of chromogenic substrate was then dispensed to each well. The reaction was stopped after 6 min by adding 50 μl of 25% (v/v) acetic acid (Sigma­Aldrich). Absorbance was read at 405 nm by a SPECTROstar® ^Nano^ (BMG Labtech, Ortenberg, Germany) plate reader. A calibration curve was included by preparing dilution series of *E. coli* 0111:B4 endotoxin (0.01–1 EU/ml). Eventually, the endotoxin concentration of filgrastim products was determined from their absorbance by linear regression. Additionally, to verify the lack of product inhibition, dilutions of filgrastim products were spiked with a known amount of endotoxin, i.e., 0.4 endotoxin units (EU)/ml. All reagents and materials used were purchased at Lonza and were endotoxin free grade-certified, unless indicated otherwise.

### Flow Imaging Microscopy

The analysis of subvisible particles was performed by flow imaging microscopy on a Micro-Flow Imaging (MFI) instrument (MFI­5200, Protein Simple, California, USA) equipped with a silane-coated 100-μm flow cell. The MFI instrument was operated at high magnification (14×) and controlled by using the MFI View System Software (MVSS) version 3.1. Between each measurement, the MFI system was flushed with 2 ml Milli-Q water at a flow rate of 0.7 ml/min and checked for a clean background. Prior to evaluating each sample, buffer components of Neupogen filtered through a 0.2-μm filter (Sartorius Stedim) was used to perform “optimize illumination” at a flow rate of 4 ml/min. Due to limited volume, a few products were analyzed only once at a sample volume of 500 μl. Data analysis was performed in MFI View Analysis Suite version 1.4. To discriminate silicone oil droplet-like particles from protein-like particles and to exclude the air bubbles from analysis, we applied filters, such as Aspect Ratio set as ≥0.87 and Intensity Min ≥20; particles with Aspect Ratio < 0.87 and Intensity Min ≥20 were considered protein-like particles (29). All chemicals were purchased from Sigma­Aldrich.

### ***In Vitro*** Potency Assay

An *in vitro* potency assay was performed based on the proliferation of M-NFS-60 cells induced by G-CSF, as described in the Ph. Eur. monograph for filgrastim concentrated solution (28). In short, all filgrastim products and the World Health Organization (WHO) 2nd International Standard (IS) for human G­CSF (NIBSC, Hertfordshire, UK) were prepared in assay medium (RPMI 1640 supplemented with L-glutamine, sodium bicarbonate, 10% fetal bovine serum, 10 mM HEPES buffer and 0.05 mM 2­mercaptoethanol) at a starting concentration of 20 ng/ml and were added to a well of a 96-well microtiter plate (Greiner Bio-One, Alphen a/d Rijn, The Netherlands) in triplicate. A series of 17 threefold dilutions was subsequently prepared to obtain a standard curve. M-NFS-60 cells were harvested and washed twice in assay medium. Hereafter, ~20,000 cells were added to each well.

After being incubated for 44–48 h at 37°C with 5% CO_2_, the cell proliferation was quantified. CellTiter 96® AQueous One Solution Cell Proliferation Assay solution (Promega, Leiden, The Netherlands) was added to the cells and the plate was re-incubated for 4 h. The quantity of formazan produced was estimated by recording the absorbance at 490 nm and 650 nm (reference wavelength) with a SPECTROstar Nano microplate reader (BMG Labtech, Ortenberg, Germany). *In vitro* potency was calculated based on WHO 2nd IS with an assigned value for G-CSF activity of 95,000 IU/ampoule using the parallel line assay in Combistats software version 5.0 (EDQM) (30).

## Results

Prior to characterization, all filgrastim products were examined visually. All products were clear and colorless solutions and contained no visible particulates.

### Structural Analysis of Filgrastim Products

**Fig. 1:**
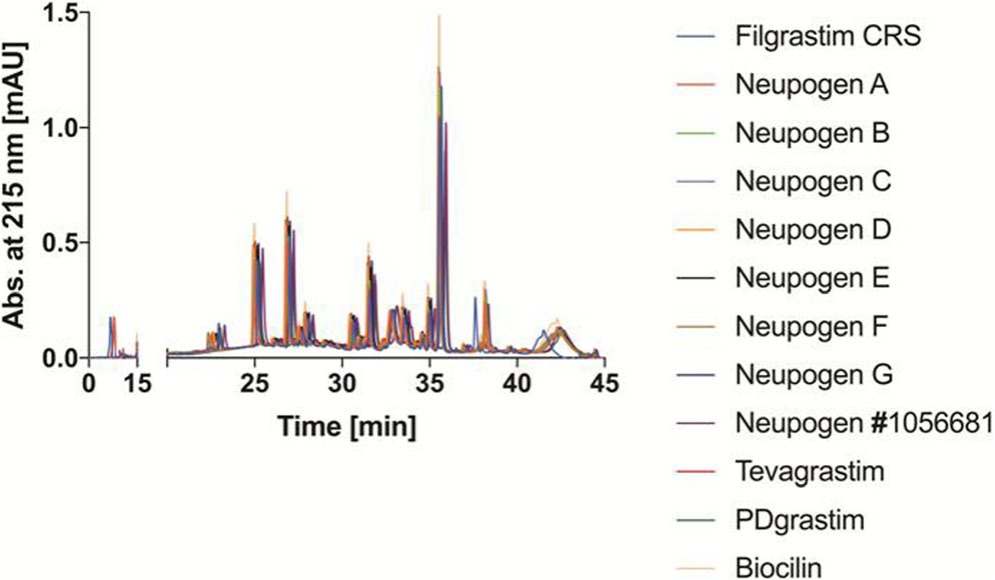
Overlay of RP-HPLC chromatograms of endoproteinase Glu-C digested of Filgrastim CRS, Neupogen batch 1056681, Tevagrastim, copy products and several batches of Neupogen.

RP-HPLC chromatograms of a Glu-C peptide map from innovator as well as selected biosimilar and copy filgrastim products are shown in Fig. 1. No differences in amino acid sequence and disulfide-bond formation were identified.

The secondary and tertiary structure of selected filgrastim products were evaluated in function of their thermal stability by circular dichroism and intrinsic fluorescence spectroscopy, respectively.

**Fig. 2:**
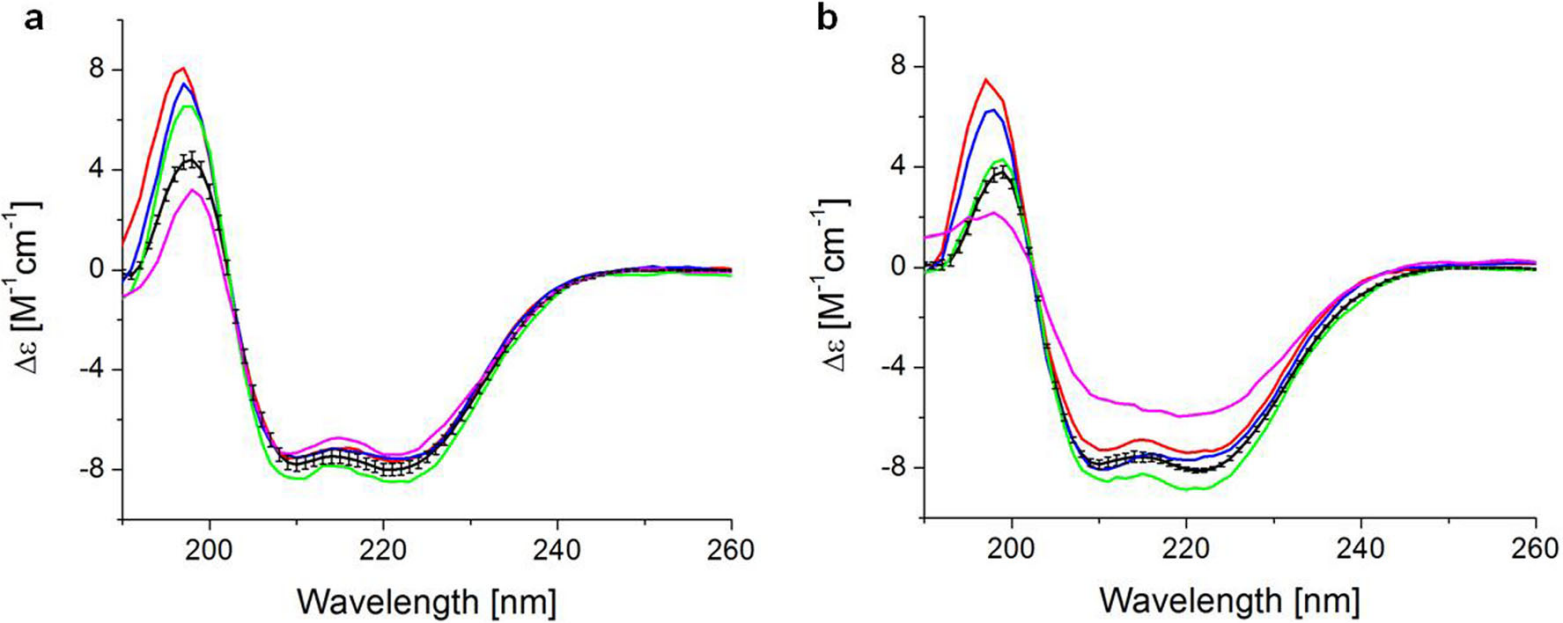
Circular dichroism spectra of filgrastim products at (a) 25 and (b) 55°C. Average curve represents the spectra of the expired bathes of Neupogen (black line), Neupogen batch 1056681 (red line), Tevagrastim (blue line), Biocilin (green line) and PDgrastim (pink line).

Fig. 2 shows the far-UV CD spectra of filgrastim products obtained at 25°C and 55°C. All products showed a positive band at 193 nm and negative bands at 208 nm and 222 nm (31), indicating they all share an alpha-helix-rich structure which has been described for filgrastim. (32–34).

Our results are consistent with those reported for other filgrastim formulations available in India (24) and Australia (35). Due to scarce information about the excipient concentrations, the differences in amplitude in the band around 193 nm could be related to the use of only one background for all samples. Except for PDgrastim, all products displayed similar thermal stability (Fig. 2b).

**Fig. 3:**
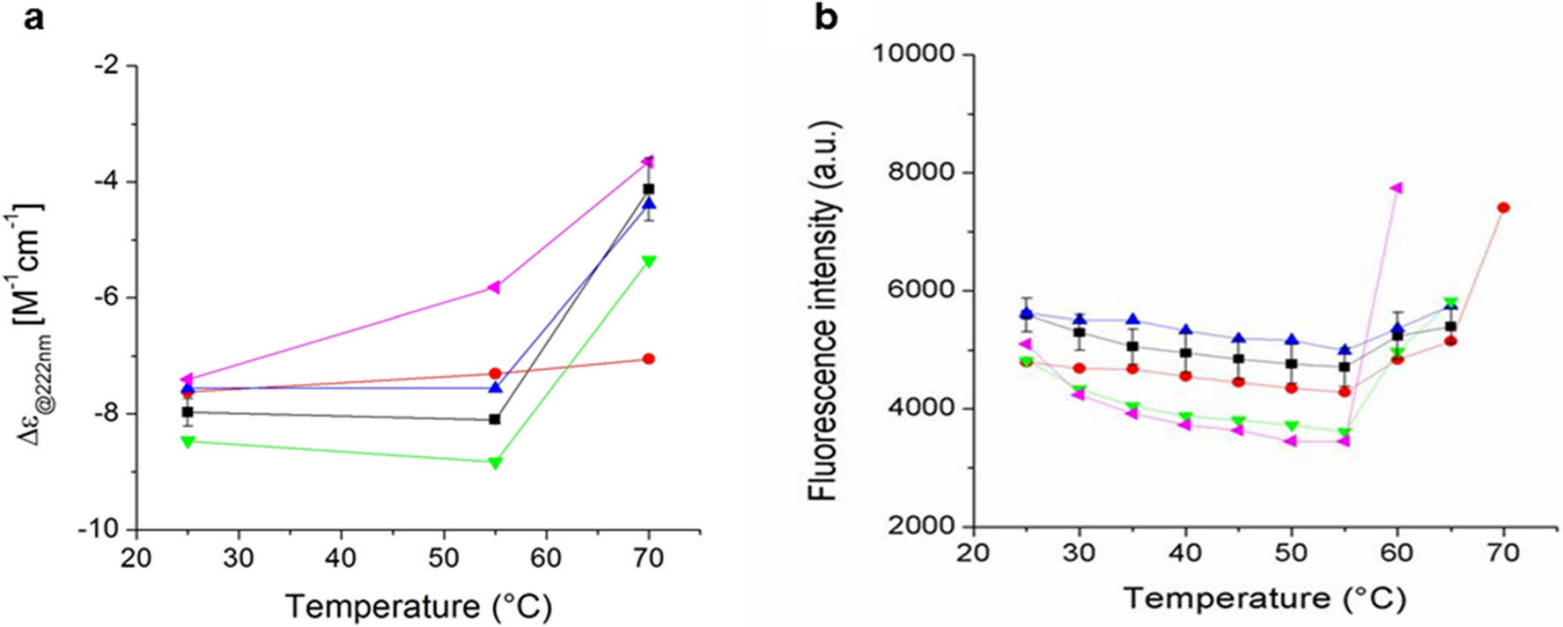
Thermal stability of selected filgrastim products monitored by (**a**) circular dichroism at 222 nm and (**b**) intrinsic fluorescence from 25 to 70°C. Average curves represent the results for expired batches of Neupogen (black line), Neupogen batch 1056681 (red line), Tevagrastim (blue line), Biocilin (green line) and PDgrastim (pink line).

Protein aggregation represents a main challenge for biopharmaceuticals, where temperature could be a factor. To assess differences in protein conformation and thermal stability between the filgrastim products, a melting curve was monitored from 25°C to 70°C by the CD signal at 222 nm and intrinsic (tryptophan and tyrosine) fluorescence intensity (Fig. 3). The filgrastim products were analyzed at low pH and the temperature of unfolding was similar in all products between 55 and 60°C. These results are in agreement with the T_m_ previously reported (33,34,36) and consistent with those reported for other filgrastim formulations (24,35). PDgrastim (pink line) showed the lowest thermal stability with a drastic change in conformation at 55°C. It is clearly different from Neupogen (red line) and the expired batches of Neupogen (black line), and consistent with the CD spectrum showing a loss of the alpha-helix structure (Fig. 2, panel b, pink line).

### Characterization of Impurities by SDS-PAGE and HP-SEC

**Fig. 4:**
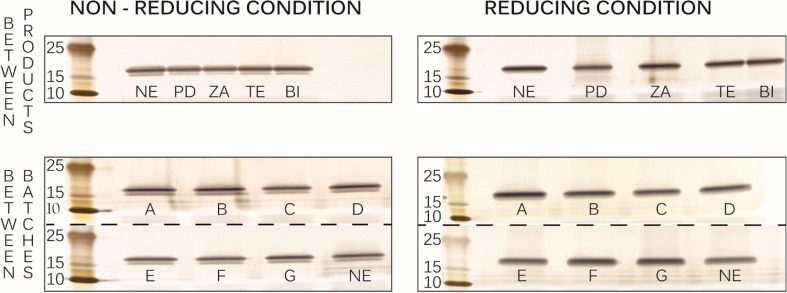
Filgrastim products on 4–12% Bolt® Bis­Tris gel visualized by silver staining. Neupogen (NE) batch 1042036A, PDgrastim (PD), Zarzio (ZA), Tevagrastim (TE) and Biocilin (BI) as well as additional batches of Neupogen (A-G).

The identity and purity of different filgrastim products were assessed by SDS-PAGE and HP-SEC. Fig. 4 exhibits silver stained SDS-PAGE gels of all products with a principal band in the range of 15–25 kDa under both non-reducing and reducing conditions. As is the case with the control (image not shown), this band corresponds to the monomeric filgrastim with a theoretical molecular weight of 18.8 kDa (19). No other bands at higher or lower molecular weight than filgrastim monomer were detected in any of the products, including the expired ones. Faint bands identified in some lanes at between 10 and 15 kDa were likely due to overloading of pre-stained protein marker.

**Fig. 5:**
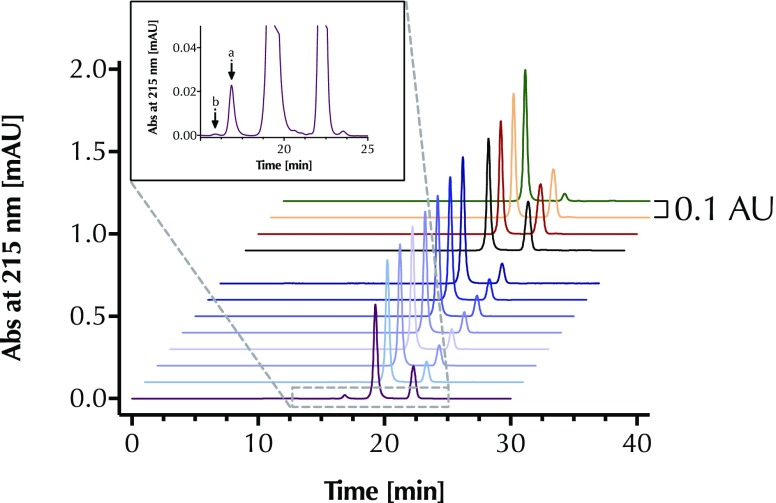
HP­SEC chromatograms of (top to bottom) Biocilin (green), Tevagrastim (pink), Zarzio (brown), and Neupogen batch 1042036A (black) followed by seven batches of expired Neupogen (several shades of blue) and PDgrastim (purple). The inset is the zoom of PDgrastim’s chromatogram. Peak (a) is filgrastim dimer and peak (b) is filgrastim oligomer.

Similarly, HP-SEC revealed no impurities, i.e., aggregates and fragments, in nearly all products including the expired batches of Neupogen (Fig. 5). For all samples the filgrastim monomer peak eluted at 19.3 min, similar to the retention time of filgrastim stated in the Ph. Eur. monograph for filgrastim concentrated solution (28). The peak eluting at 23 min is derived from components of the dilution buffer.

In the case of expired products, only in PDgrastim (inset Fig. 5), we detected filgrastim dimer (eluting at 16.9 min) and oligomer (eluting at 16.0 min) based on relative retention with reference to the filgrastim monomer stated in the Ph. Eur. monograph for filgrastim concentrated solution (28). The relative peak area representing dimer and oligomer compared to the total area of all protein peaks in the chromatograms was found to be ~3.6% and ~0.1%, respectively. The relative peak area of dimer was found to be higher than the limit of 2% stated in the Ph. Eur. monograph for filgrastim concentrated solution (28).

As summarized in Table II, we used HP-SEC to measure the monomer content of all filgrastim products based on a calibration curve with filgrastim CRS where the monomer peak areas were plotted against the concentration of filgrastim CRS. Linearity was observed in a concentration range from 0.05 to 1 mg/ml (R^2^ = 0.9975). Both Tevagrastim and Zarzio showed a filgrastim monomer content comparable to that of Neupogen. In contrast, the monomer content of Biocilin and PDgrastim were more than twofold lower than that of Neupogen. Moreover, the expired batches of Neupogen showed a substantially reduced monomer content. Additionally, the ratio between the total peak areas of filgrastim monomer detected by fluorescence and UV detectors was a bit lower for PDgrastim as compared to all other products. This may point to a difference in protein structure and aggregation as shown by HP-SEC, although we cannot exclude that PDgrastim has shown different stability by fluorescence spectroscopy than the rest of the products.

**Table II:** Comparison of Monomer Content and Potency of Tested Filgrastim Products

Trade Name		Monomer content HP­SEC, UV_215 nm_ [mg/ml] ± SD	*In vitro* potency [Lower-Upper limit]	Total AUC fluorescence / total AUC UV_280 nm_ from HP-SEC ± SD	Specific activity
Neupogen		0.647 ± 0.022	24.1 MU/0.5 ml [23.1–25.1]	719.2 ± 6.6	74.4 MU/mg
Zarzio		0.650 ± 0.028	30.3 MU/0.5 ml [29.1–31.6]	737.6 ± 5.1	93.2 MU/mg
Tevagrastim		0.691 ± 0.027	31.1 MU/1.0 ml [29.8–32.4]	735.4 ± 6.9	89.9 MU/mg
Biocilin		0.317 ± 0.016	23.1 MU/1.2 ml [22.2–24.1]	732.4 ± 22.9	60.7 MU/mg
Neupogen	A	0.360 ± 0.017	40.5 MU/1.6 ml [38.9–42.2]	730.9 ± 27.0	70.3 MU/mg
	B	0.353 ± 0.016	39.8 MU/1.6 ml [38.2–41.5]	741.6 ± 8.0	70.4 MU/mg
	C	0.358 ± 0.016	41.6 MU/1.6 ml [39.9–43.3]	747.0 ± 21.8	72.6 MU/mg
	D	0.354 ± 0.016	42.1 MU/1.6 ml [40.4–43.8]	737.4 ± 27.2	74.3 MU/mg
	E	0.353 ± 0.017	41.1 MU/1.6 ml [39.4–42.8]	737.3 ± 11.1	72.7 MU/mg
	F	0.359 ± 0.016	39.3 MU/1.6 ml [37.7–40.9]	738.1 ± 11.4	68.4 MU/mg
	G	0.368 ± 0.015	41.9 MU/1.6 ml [40.2–43.7]	729.4 ± 17.0	71.2 MU/mg
PDgrastim		0.258 ± 0.007	26.4 MU/0.5 ml [25.4–27.5]	669.1 ± 27.9	102.4 MU/mg

*SD* standard deviation

### Identification of Related Proteins of Filgrastim

The oxidation of methionine residues to methionine sulfoxide or methionine sulfone is one of the degradation pathways present in biopharmaceuticals during formulation and storage. Common antioxidants used are glutathione, acetylcysteine, methionine, ascorbic acid and sodium bisulfite (37). However, filgrastim formulations do not contain an antioxidant (24), maybe due to low oxidation rate of Met1, Met122, Met127, Met138 at low pH (37).

**Fig. 6:**
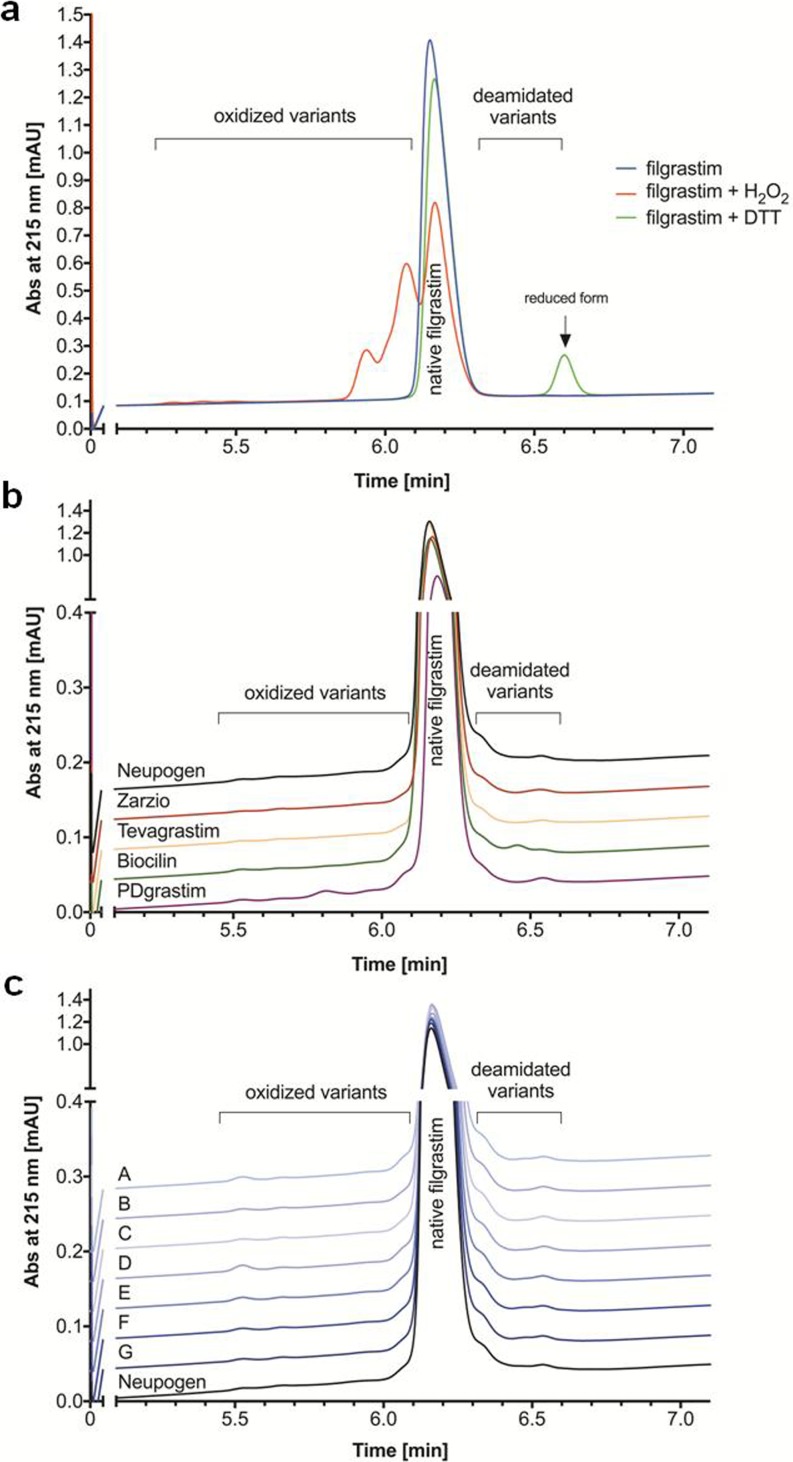
RP-UPLC chromatograms of (**a**) filgrastim CRS, (**b**) biosimilar and copy filgrastim products, and (**c**) expired batches of Neupogen compared to non-expired Neupogen.

Possible product-related impurities, such as oxidized and reduced variants, contained in filgrastim products were analyzed by RP-UPLC. As in the case with filgrastim CRS, all products showed a main filgrastim peak, which eluted at ~6.2 min (Fig. 6). Filgrastim CRS and all products showed oxidized variants, which eluted between 5.5 min and 6.0 min and deamidated variants eluted between 6.3 and 6.5 min (Fig. 6a). While PDgrastim contained distinct oxidized variant at 5.8 min, Biocilin contained another deamidated variant at 6.4 min (Fig. 6b). The total area percentage of either oxidized or deamidated variants was calculated for each filgrastim product and compared to the total peak area of all peaks in the chromatogram. The relative peak area of either oxidized or deamidated variants for Zarzio and Tevagrastim was below the limits stated on Ph. Eur. (28) and were comparable to those of Neupogen. While the relative peak area of the impurity in Biocilin was below the limits, PDgrastim contained a relative peak area of oxidized variants ~2.1% above the acceptable limit.

Oxidation is a common degradation pathway, often resulting in structural changes (38) and might become the shelf-life limiting factor (39). PDgrastim shows the highest level of oxidation, aggregation and loss of second and tertiary structures, maybe due to a difference in excipients. Expired batches of Neupogen showed comparable low relative peak areas of oxidized and deamidated variants as the non-expired batch (Fig. 6c).

### Evaluation of Bacterial Endotoxin in Filgrastim Products

Endotoxin may be introduced during manufacturing especially in the case of filgrastim as it is expressed in *E. coli*. Hence, it is critical to quantify endotoxin in drug products because the presence of endotoxin can result in pyrogenic responses and may affect immunogenicity of the finished products (40,41). The endotoxin level of Biosimilars (Zarzio and Tevagrastim) and copy products (Biocilin and PDgrastim) was compared with the innovator (Neupogen batch 1042036A and expired Neupogen A-G). The endotoxin content of all filgrastim products was far below the limit 2 IU/mg of protein, as stated in the Ph. Eur. monograph on filgrastim concentrated solution (28).

### Characterization of Subvisible Particles in Filgrastim Products

**Fig. 7:**
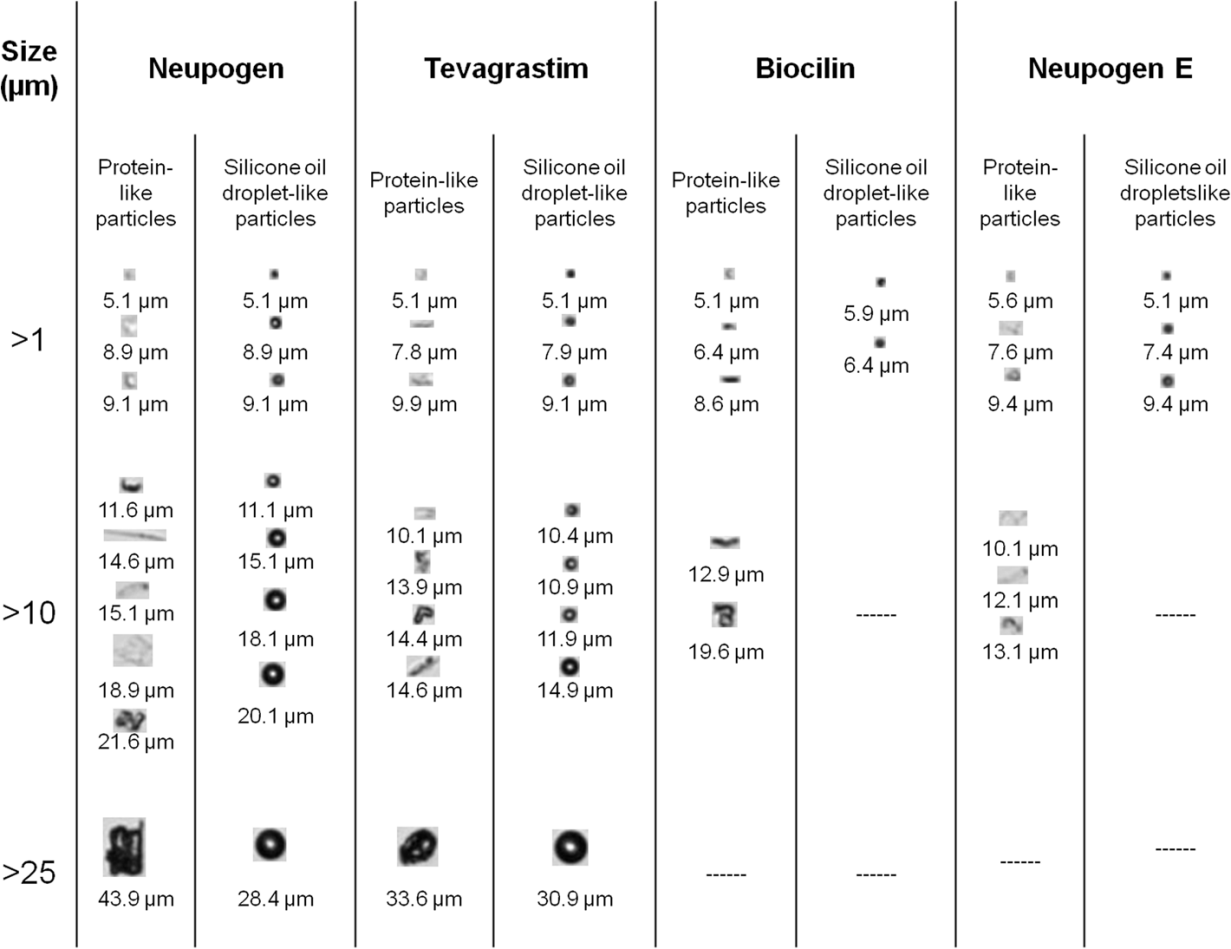
Representative images of protein-like and silicone oil droplet-like particles observed by MFI in selected filgrastim products.

Subvisible particles in the range of 1–100 μm were sized and counted by MFI. Silicone oil droplets are distinguished from proteinaceous particles by using the image filtering capabilities of MFI, namely aspect ratio and intensity minimum, as described elsewhere (29). Fig. 7 exhibits representative images of protein-like and silicone oil droplet-like particles. In contrast to the uniform circularity of the silicone oil droplets, protein-like particles are highly heterogeneous in shape and size. Filgrastim proteinaceous particles ranged from small translucent ovals of 5 μm to fiber-like and irregularly shaped particles up to about 40 μm.

The particle concentrations are listed in Table III. Neupogen and Tevagrastim supplied in PFS had much higher concentrations of protein-like and silicone oil droplet-like particles than the other analyzed products, which are presented in vials. In contrast with the glass vial with rubber stopper, the PFS has multiplex reactive surfaces which may contribute to the formation of particles (42,43). Considering that MFI is more sensitive than light obscuration to detect proteinaceous particles (29), our results suggest that in all cases, the average number of particles present in the analyzed filgrastim products was far below the limit of 6000 particles ≥10 μm/container and 600 particles ≥25 μm/container based on light obscuration, as stated in the respective Ph. Eur. monograph (44).

**Table III:** Comparison of Protein-Like Particles and Silicone Oil Droplet-Like Particle Concentrations (Particles/ml) in Some Filgrastim Products Measured by MFI

Trade name		Size range [μm]									
		Protein-like particles [particles/ml]					Silicone oil droplet-like particles [particles/ml]				
		≥1	≥2	≥5	≥10	≥25	≥1	≥2	≥5	≥10	≥25
Neupogen		60,369	18,244	764	92	4	17,724	25,123	4945	363	11
Tevagrastim		27,439	4276	237	38	4	6932	3638	397	57	4
Biocilin		8021	546	23	8	0	2182	413	8	0	0
Neupogen	A	9260	944	57	8	4	2797	1620	134	4	0
	B	15,557	1972	164	69	0	6722	3183	88	11	0
	E	19,440	1525	103	27	0	6168	2174	118	0	0
	F	20,873	1204	99	11	8	6199	1380	88	19	4

### Determination of *In Vitro* Potency of Filgrastim Products

The *in vitro* potency of filgrastim products was measured by comparing its proliferative effect in M-NFS-60 cells with 2nd WHO G-CSF IS. Table II lists the estimated potencies of all products. The *in vitro* potency of most products was between 80 and 125% of the declared potency, thereby meeting the specifications of the Ph. Eur. monograph for filgrastim concentrated solution (28). However, for Biocilin this could not be determined because the package or website does not state the potency.

Specific activity was determined based on the outcome of the potency assay and the filgrastim monomer content. We focused on the monomer due to higher *in vitro* biological activity than filgrastim dimers (45). Whereas Tevagrastim and Zarzio showed a slightly higher specific activity than Neupogen, the specific activity of PDgrastim was substantially higher. In contrast, Biocilin exhibited a lower specific activity than Neupogen. All expired batches of Neupogen had a comparable specific activity as the non-expired batch.

## Discussion

The current study showed that the quality of analyzed filgrastim products approved by the EMA (Zarzio and Tevagrastim) was comparable to that of the innovator product (Neupogen), whereas the copy filgrastim products (Biocilin and PDgrastim) differed substantially in specific activity and especially PDgrastim also showed other differences in quality.

While the stated filgrastim concentration of Biocilin (0.25 mg/ml) is 2.4-fold lower than that of Neupogen (0.6 mg/ml), we calculated a higher filgrastim monomer content of Biocilin (0.32 mg/ml) as is evident from HP-SEC. On the other hand, Biocilin showed a lower specific activity compared to Neupogen. Although the reason for this remains speculative, a lower specific activity may indicate the presence of impurities or denatured protein. Mexico was one of the first countries in Latin America to introduce a biosimilar regulatory pathway in 2009, Biocilin was introduced in Mexico before the biosimilar guidelines were put in place (46). Data on quality and biological activity are not available in the public domain. In contrast, the physicochemical and biological characterization of Zarzio is available and shows a high similarity in the primary, secondary and tertiary protein structure, mass, size, purity, charge, hydrophobicity and bioactivity to Neupogen (18). However, a recent pharmacovigilance study conducted in Mexico for filgrastim products including Biocilin in cancer patients detected no new adverse events, suggesting that the observed quality differences in Biocilin seem not to have any impact on product safety (47). PDgrastim and several batches of Neupogen were received close to their expiration date. Even so we included them, to gain insight into the quality of expired copy filgrastim product in comparison with expired innovator as a worst case scenario in the market. Although PDgrastim displayed a lower monomer content than stated and 2.5-fold lower than Neupogen, it exhibited a substantially higher specific activity compared to Neupogen. This could be due to differences in structure, as demonstrated by HP-SEC. In addition to specific activity, PDgrastim also showed a higher relative content of dimer and oxidized variants, and a lower thermal stability than Neupogen. No differences were observed in any expired batches of Neupogen.

We observed a higher concentration of silicone oil droplet-like particles of >10 and > 25 μm in products supplied in PFS (Neupogen and Tevagrastim). The pre-filled syringes use silicone oil to lubricate the needle and glass barrel to improve the motion of the plunger (42,48).

We analyzed two copy filgrastim products, but it should be considered that more products are available in less regulated markets. Testing these products will provide more insight into the quality of available copy filgrastim products in general. Additionally, future studies should include more assays and batches to provide more robust comparative quality data. Here, we clearly show that there is not excuse to skip the physicochemical and biological comparison between copy products and innovator by any manufacturer.

## Conclusion

Here, we report a head-to-head comparative quality study of biosimilars approved in Europe and US, and copy filgrastim products available in developing countries. Using selected analytical tools and *in vitro* bioassay, we demonstrate no significant differences in quality between the filgrastim products studied, except for the specific activity of the two copy products of filgrastim. PDgrastim showed presence of impurities and lower thermal stability than the rest of the products. Looking forward manufacturers should put more efforts in making such comparative quality studies.

## Abbreviations

AIDS: Acquired Immunodeficiency Syndrome
BPCI Act: Biologics Price Competition and Innovation
CD: Circular Dichroism
CRS: Chemical Reference Substances
*E. coli*: *Escherichia coli*
EDQM: European Directorate for the Quality of Medicines and HealthCare
EU: European Union
FDA: Food and Drug Administration
FWHM: Full Width at Half Maximum
HP-SEC: High Performance Size-Exclusion Chromatography
LAL: Limulus Amebocyte Lysate
MFI: Micro­Flow Imaging
PEG: Polyethylene Glycol
PFS: Pre-Filled Syringe
Ph. Eur.: European Pharmacopeia
PS80: Polysorbate 80
rhG-CSF: Recombinant Human Granulocyte-Colony Stimulation Factor
RP-UPLC: Reversed Phase Ultra-Performance Liquid Chromatography
SDS­PAGE: Sodium Dodecyl Sulfate Polyacrylamide Gel Electrophoresis
UMCU: University Medical Centre Utrecht
US: United States
